# Imaging Features of Breast Periductal Stromal Tumor: A Case Report

**DOI:** 10.3389/fonc.2021.577227

**Published:** 2021-10-15

**Authors:** Ning Ding, Ying Jiang, Haimin Liu, Fuling Zheng, Shenling Zhu, Ming Wang, Meng Yang, Lingyan Kong, Huadan Xue, Zhengyu Jin

**Affiliations:** ^1^ Department of Radiology, State Key Laboratory of Complex Severe and Rare Diseases, Peking Union Medical College Hospital, Chinese Academy of Medical Science and Peking Union Medical College, Beijing, China; ^2^ Department of Pathology, State Key Laboratory of Complex Severe and Rare Diseases, Peking Union Medical College Hospital, Chinese Academy of Medical Science and Peking Union Medical College, Beijing, China; ^3^ Department of Medical Record, State Key Laboratory of Complex Severe and Rare Diseases, Peking Union Medical College Hospital, Chinese Academy of Medical Science and Peking Union Medical College, Beijing, China; ^4^ Department of Ultrasound, State Key Laboratory of Complex Severe and Rare Diseases, Peking Union Medical College Hospital, Chinese Academy of Medical Science and Peking Union Medical College, Beijing, China

**Keywords:** breast periductal stromal tumor, imaging, ultrasonography, mammography, digital breast tomosynthesis

## Abstract

Breast periductal stromal tumor (PDST) is a rare biphasic tumor, with both benign ductal epithelium and non-phyllodes sarcomatous stroma. Its imaging features were rarely reported due to the rarity. In this study, we describe the case of a 48-year-old female who presented with a palpable mass in the right breast. Presurgery imaging evaluations of full-field digital mammography (FFDM), digital breast tomosynthesis (DBT), and ultrasonography (US) were performed. The imaging features include the following: 1. multiple solid lobulated lesions comprising nearly the entire right breast; 2. hypoechoic heterogeneous masses with internal separations and abundant blood flow; 3. FFDM and DBT showed multiple irregular high-density masses with lobulated margin, partially integrated. The patient underwent extended mastectomy of the right breast. The surgical pathology confirmed a PDST. After excision of the mass, she was followed up in the outpatient clinic for 25 months without local recurrence or distant metastasis.

## Introduction

Breast periductal stromal tumor (PDST) is a rare condition and accounts for less than 1% of all breast malignancies. As far as we know, only sporadic cases were reported (1–9). As a biphasic tumor, PDST shows both benign ductal epithelium and non-phyllodes sarcomatous stroma. It is most likely to originate from the periductal stroma (10). Considering the infiltrative characteristic of the tumor, surgical excision with enough margin is required (6). Presurgery imaging plays an important role in the surgical planning and the follow-up management of PDST. However, because of the rarity of the disease, its radiological features were seldom reported. We present a case of breast PDST with multiple-modality preoperational images, including full-field digital mammography (FFDM), digital breast tomosynthesis (DBT), and ultrasonography (US).

## Case Presentation

Written informed consent was obtained from the individual for the publication of any potentially identifiable images or data included in this article.

**Figure 1:**
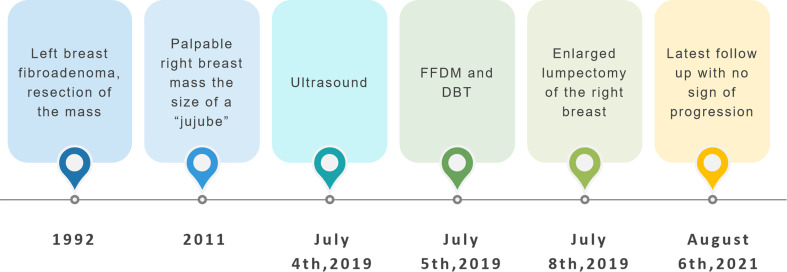
Timeline of the relevant data of this case.

A 48-year-old woman was admitted with an 8-year history of right breast mass. The mass had been steady at the size of a “jujube” until it grew rapidly during the few months before admission. The patient, with no family history of breast cancer, had a history of fibroadenoma excision of the left breast 27 years ago. On physical examination, her right breast was grossly plumper than the opposite, with an approximately 8-cm palpable mass, which was painless and mobile. Remarkable subcutaneous varicose veins were shown in her right breast. Nipple discharge was not reported (**Figure 1**).

**Figure 2:**
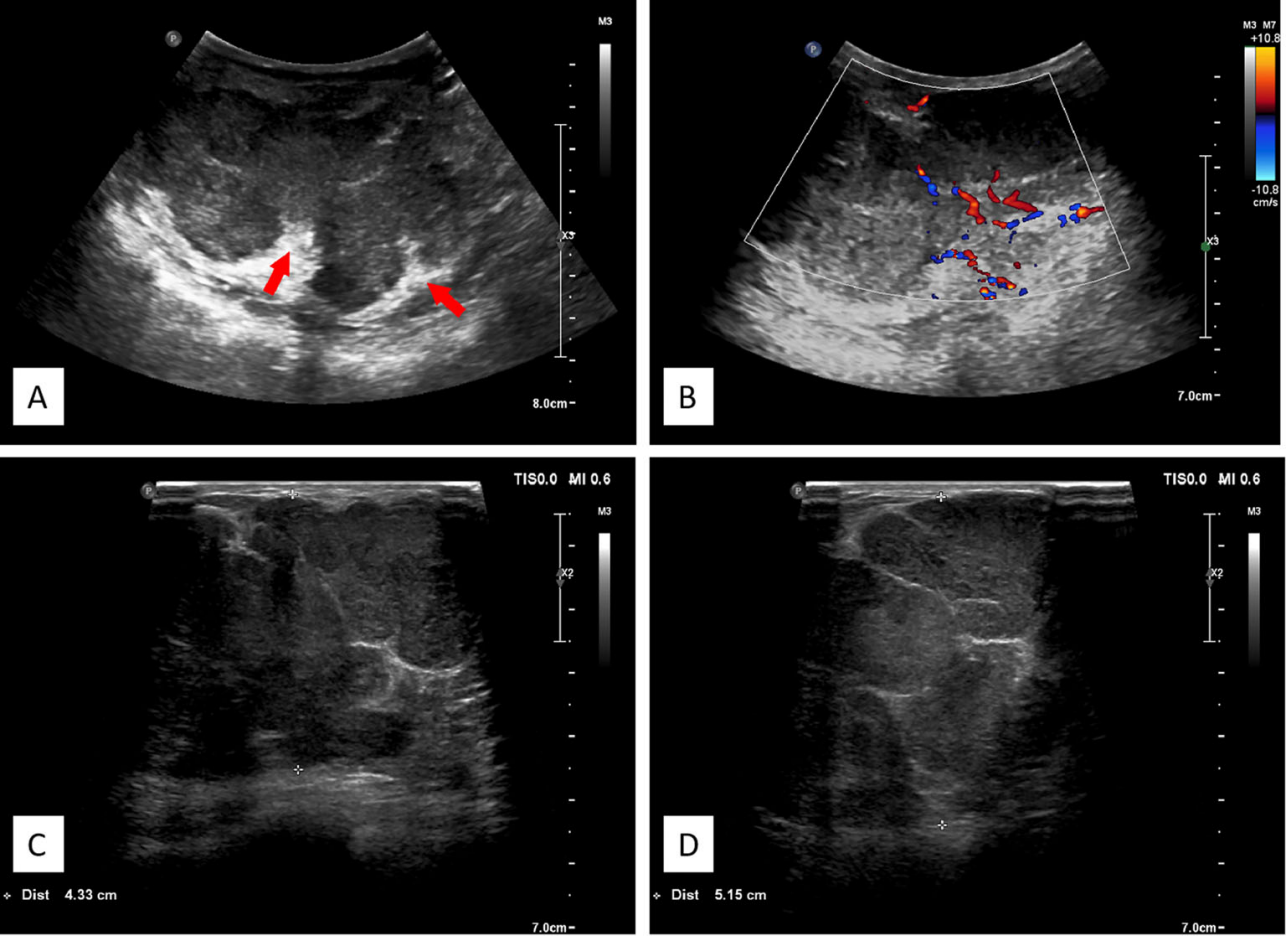
**(A)** Ultrasonographic image showing multiple hypoechoic lesions in the right breast. The large masses comprising nearly the entire right breast, with lobulated irregular margin, the banded median-high echogenic septum was seen inside the mass. **(B)** Color flow Doppler image showing abundant blood flow signals. **(C, D)** Different spliced images of the tumor showed irregular margin with multiple septa.

A 3–12-MHz linear transducer (RS80A with Prestige, Samsung Medison, Co. Ltd.) was utilized for the US examination. The initial US revealed multiple irregular hypoechoic masses with lobulated irregular margin (red arrow) in the right breast. The largest lesion was located 2 cm from the areola area, which measured 7.1 cm × 9.1 cm × 5.6 cm, and internal mass echogenicity was heterogeneous with abundant internal vascularity. Multiple banded hyperechogenic septa were noted (**Figure 2**).

**Figure 3:**
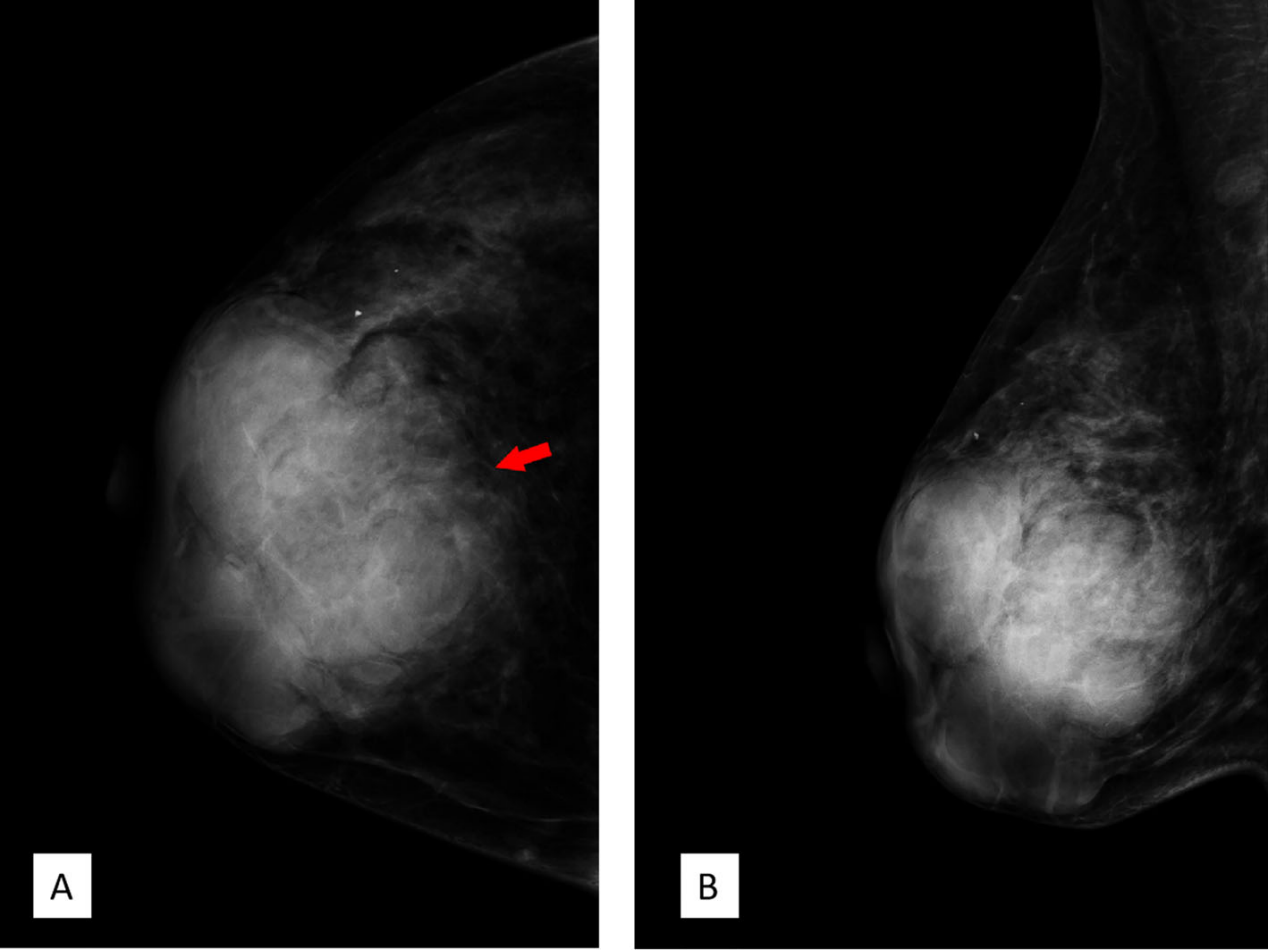
Full-field digital mammography. **(A)** Craniocaudal position and **(B)** mediolateral oblique position showed multiple high-density irregular masses, some of which were integrated with obscured septa, with multiple lobulations. Note the red arrow in panel A; the margin of the lesion was obscured by surrounding structures. Breast Imaging-Reporting and Data System (BI-RADS) 3.

FFDM (Senographe Essential, General Electric Company, USA) revealed integrated multiple irregular lobulated high-density masses comprising nearly the entire right breast that is asymmetrically enlarged compared to the left. There is no suspicious finding in the left breast (**Figure 3**).

**Figure 4:**
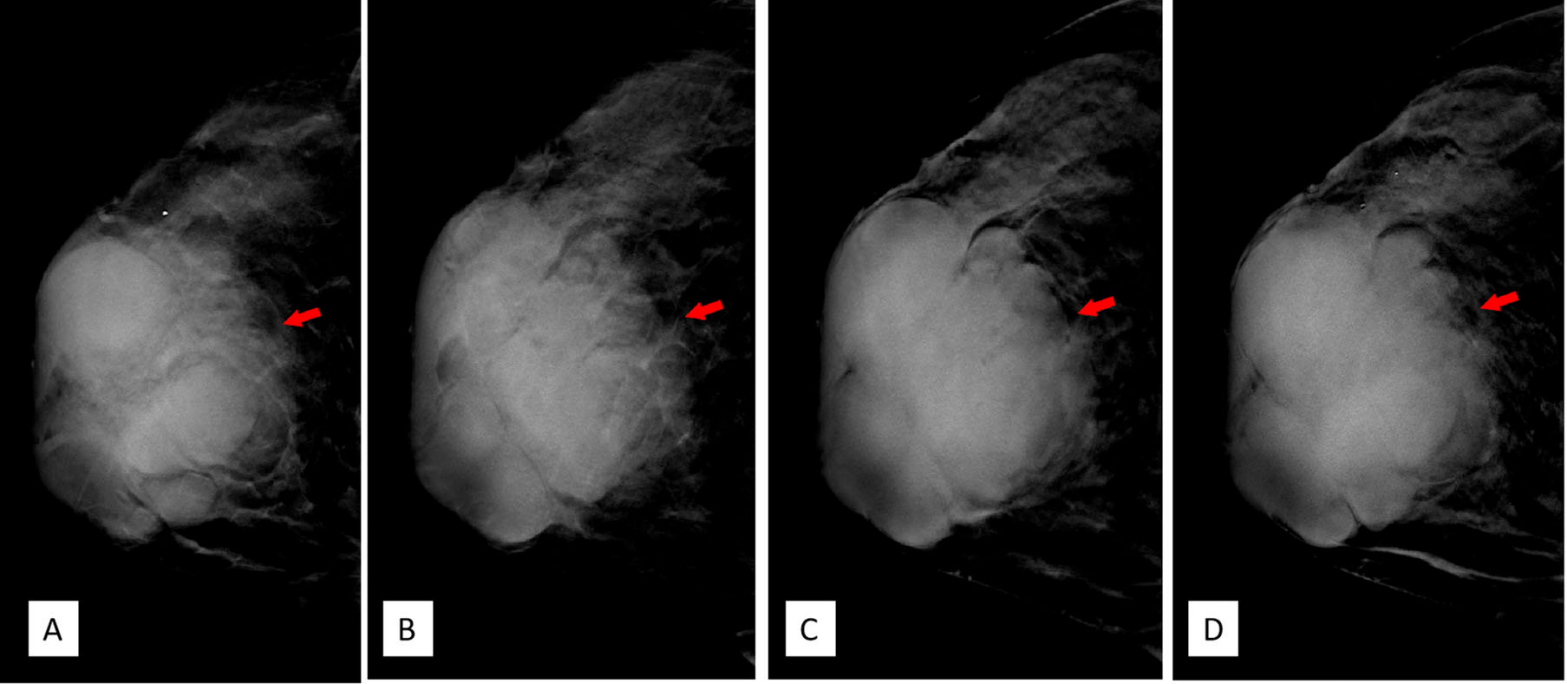
**(A–D)** Craniocaudal position of digital breast tomosynthesis. The red arrow shown in panels **(A, B)** demonstrated that the margin of the mass was obscured; however, the red arrow area in panels **(C, D)** showed unshielded margin of the mass, which was more obvious compared with that in **Figure 3A**.

**Figure 5:**
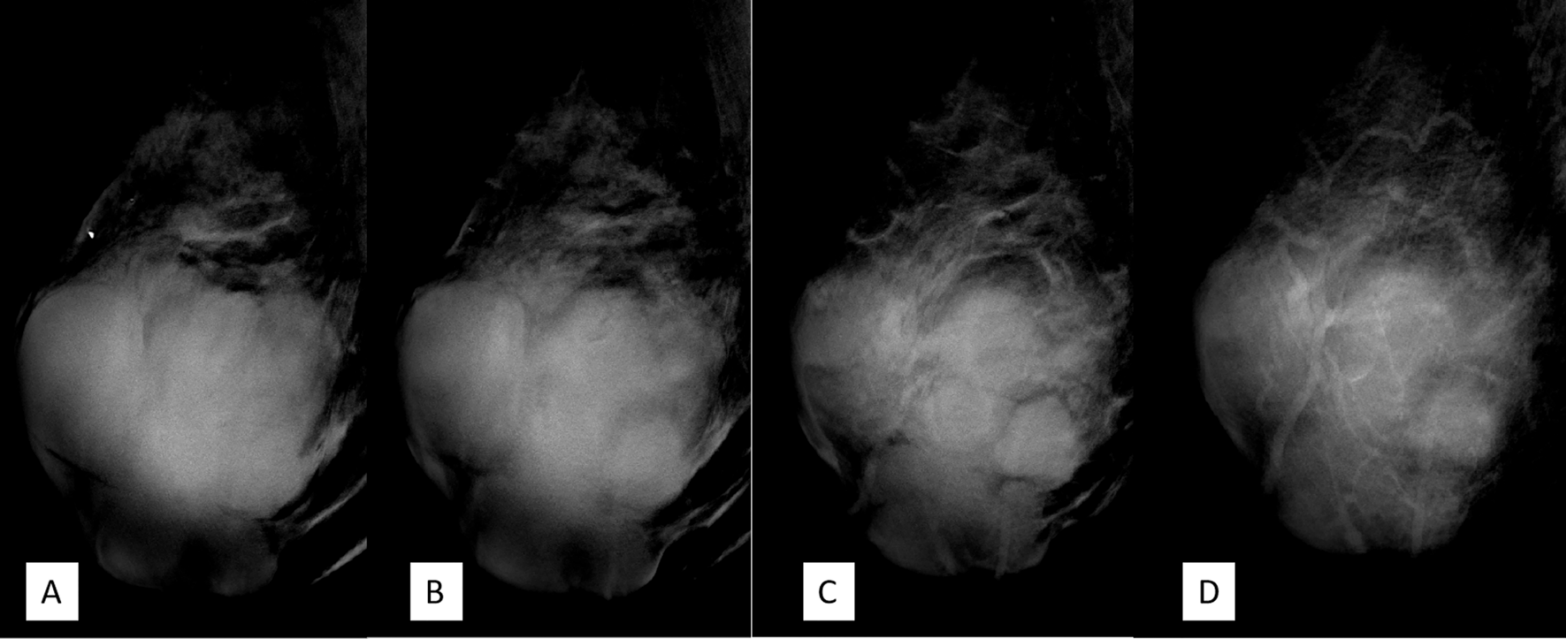
**(A–D)** The mediolateral oblique position of digital breast tomosynthesis demonstrated improvement in sharpness and internal septa of the large mass; it measured 7.8 cm × 9.1 cm × 7.4 cm in size, with irregular margins and multiple lobulations. Breast Imaging-Reporting and Data System (BI-RADS) 4A.

DBT (Senographe Essential, General Electric Company, USA) demonstrated improvement in sharpness of the margins and internal septa of the masses. The masses measured in total 7.8 cm × 9.1 cm × 7.4 cm in size, integrated, with irregular shape and multiple lobulations. No enlarged lymph nodes were noted (**Figures 4**, **5**).

The breast lesion was classified as Breast Imaging-Reporting and Data System (BI-RADS) 3 by FFDM and BI-RADS 4A based on DBT and US. Considering the size and the rapid growth of the mass, surgical excision was recommended without biopsy.

The patient underwent extended mastectomy of the right breast. After the surgery, she received neither chemotherapy nor radiotherapy. She is currently doing well without any sign of recurrence for 25 months.

**Figure 6:**
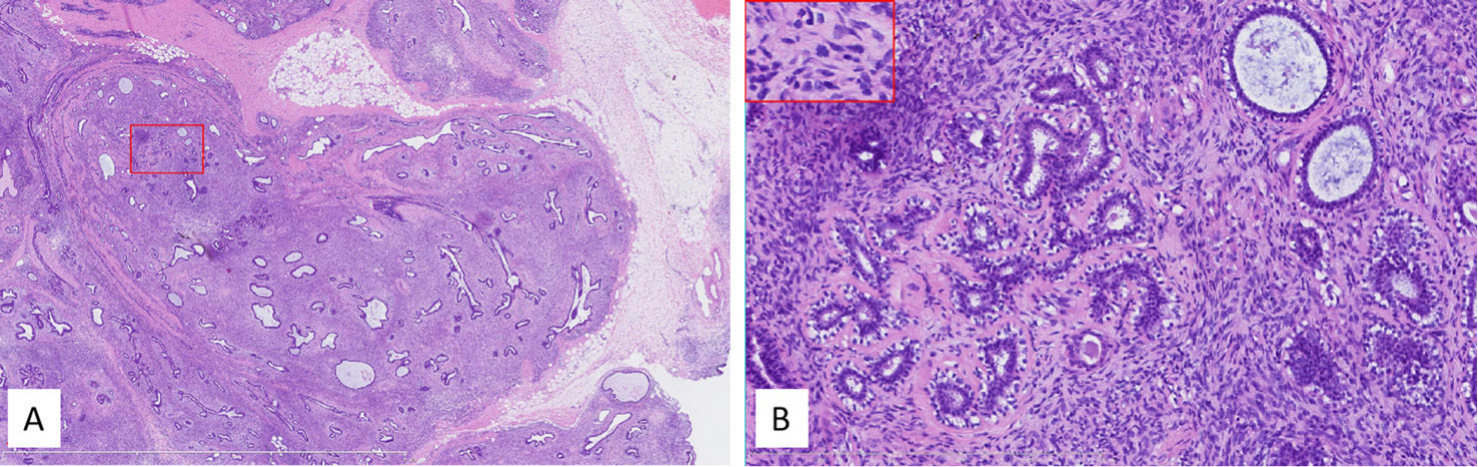
**(A)** Low power, the tumor lacked obvious leaflike processes while it partially retained the lobular architecture. The tumor infiltrated peripheral tissues, including fat (right middle margin). The red box indicated the corresponding area shown in panel **(B)** Internal bar = 6 mm. **(B)** Higher power showed high stromal cellularity with obvious dysplasia, active mitoses, and pathologic mitosis [middle in the insert (embraced with red box, located left upper corner)]. The ducts in the tumor kept a nearly normal contour, and the stromal cellularity adjacent to the epithelium did not obviously increase. Internal bar = 500 μm.

Grossly, the tumor in the lumpectomy specimen measured 8.5 cm × 6.5 cm × 5 cm in size with a gray-pink section, solid texture, and lobulated borders. The pathological diagnosis was a PDST of the breast. As for the determination of malignancy, this case demonstrated invasive growth, significant pleomorphism, active mitosis (>10/10 HPF or >5/mm^2^) with pathological mitosis, and was rich in mesenchymal cells, so it was classified as malignant (**Figure 6**).

## Differential Diagnoses

### Phyllodes Tumor

The imaging features of phyllodes tumor and PDST overlap. They were mainly distinguished by pathological characteristics. Unlike phyllodes tumors, PDST does not present a leaflike architecture. In our case, the tumor had no obvious phyllode fissure; however, it partially retained the structure of breast lobules. The residual breast ducts and lobules in the tumor maintained a normal contour, while the cell density at the epithelial–mesenchymal junction did not increase significantly.

### Juvenile Fibroadenoma

When the size of a fibroadenoma is greater than 5 cm with increased stromal cellularity and epithelial hyperplasia on tissue analysis, it is classified as juvenile fibroadenoma (11). Juvenile fibroadenoma is uncommon, usually seen in adolescent girls (10); hence, juvenile fibroadenoma is less likely in a patient of middle age. Fibroadenomas typically present as unilateral firm non-tender masses. However, tissue pathology is required for a definitive diagnosis.

### Invasive Ductal Carcinoma

Invasive ductal carcinoma (IDC) can be displayed as a large mass or multiple fused masses and enlarge rapidly. The histopathological features of IDC vary considerably from case to case and can even vary within the same case. However, IDC mass at this size is often associated with obvious malignancy features including infiltrative boundary and non-circumscribed margins that were not shown in this case. Moreover, the lack of risk factors makes breast carcinoma less likely.

## Discussion

The PDST has been suggested as part of the spectrum of phyllodes tumor, and the name PDST was adopted in the fourth edition of WHO Classification of Tumors of the Breast published in the year 2012 (12, 13), as these two types exhibit similar pathological features and progression from PDST to phyllodes tumor has been documented (6). In the fifth edition of WHO Classification of Tumors, PDST is distinguished with the feature lacking the fronded architecture of typical phyllodes tumors, and it is regarded as a subtype of phyllodes tumor (10). So far, the knowledge of PDST mainly comes from case reports; no standard of care has been established.

According to the largest case serial report of PDST by Burga et al. (6), PDST is more commonly seen in perimenopausal and postmenopausal women (6); however, a single case of PDST in a 14-year-old girl has also been reported (8). The reported mass size was diversified between 0.2 and 20 cm (1–6, 14–16). Surgery with negative margins is commonly accepted as the first choice of treatment. The prognosis and standard of care for PDST is not well documented. Although most phyllodes tumors are benign, PDSTs displayed varying degrees of atypia and mitotic activity and were depicted as a low-grade malignant in 2012 WHO Classification of tumors of the breast. In this case, infiltrative growth, rich in interstitial cells, obvious atypia, active mitosis (>10/10 HPF or 5/mm^2^), and pathological mitosis indicated malignancy. Recurrent rate for PDST was reported varying from 10% to 55% (7, 17). Margin status appears to be the most relevant factor for prognosis. Metastasis of phyllodes tumors to axillary lymph nodes is rare; however, for malignant cases, distant metastasis could be seen 5–8 years after diagnosis. For this case, a 3-month interval follow-up plan was recommended. Fortunately, until the very last follow-up, no sign of local recurrence or distant metastasis is noted.

Preoperational imaging provides essential information for individualized surgical planning.

As far as we know, no classic imaging features have been reported due to the rarity of PDST. Our case offered detailed imaging features on US, FFDM, and DBT of PDST. The key imaging characteristics were the following: (1) multiple solid lobulated lesions comprising nearly the entire right breast; (2) hypoechoic and heterogeneous echo patterns with internal separations and abundant blood flow were noted; (3) FFDM and DBT showed multiple irregular high-density masses with lobulated margin, partially integrated. To our knowledge, this is the first case report of PDST with descriptions of multiple imaging modalities; these findings are consistent with previous studies (1–6, 14) with informative morphological details.

DBT, which involves multiple projections, is a promising technique for breast cancer screening and diagnosis. Compared with FFDM alone, DBT plus FFDM demonstrated higher cancer detection rate (18). Moreover, DBT showed improvements in lesion detection, characterization, and localization. In this case, the suspicious feature of multiple internal septa and irregular boundaries were more clearly depicted in DBT than FFDM (**Figures 3** **–** **5**), providing more evidence for a precise diagnosis.

## Conclusion

We provided a case of PDST with imaging of FFDM, DBT, and the US. The detailed imaging feature of the PDST case was depicted. DBT was recommended than FFDM alone as the added value in recognizing suspicious features. Presurgery imaging can provide general information on breast mass, making it easier to formulate surgical and follow-up plans.

## Data Availability Statement

The original contributions presented in the study are included in the article/supplementary material. Further inquiries can be directed to the corresponding authors.

## Ethics Statement

The studies involving human participants were reviewed and approved by the Institutional Review Board (IRB) of Peking Union Medical College Hospital (PUMCH) that has reviewed the study. The patient/participant provided her written informed consent to participate in this study. Written informed consent was obtained from the individual for the publication of any potentially identifiable images or data included in this article.

## Author Contributions

Guarantor of integrity of the entire case study: LK, ZJ, HX. Study concepts and design: LK, FZ,ND, YJ, HL, MW, MY, FZ, SZ. Literature research: All authors. ND, MY, SZ, HX, LK, ZJ. Imaging analysis: SZ, MW, MY, YJ, HL, FZ, ND, LK. Manuscript preparation: ND, LK. All authors contributed to the article and approved the submitted version.

## Funding

This research was funded by the National Key R&D Program of China (No. 2017YFC1309100) and the National Public Welfare Basic Scientific Research Program of Chinese Academy of Medical Sciences (2018PT32003 and 2017PT320004).

## Conflict of Interest

The authors declare that the research was conducted in the absence of any commercial or financial relationships that could be construed as a potential conflict of interest.

## Publisher’s Note

All claims expressed in this article are solely those of the authors and do not necessarily represent those of their affiliated organizations, or those of the publisher, the editors and the reviewers. Any product that may be evaluated in this article, or claim that may be made by its manufacturer, is not guaranteed or endorsed by the publisher.
